# Vimentin – Force regulator in confined environments

**DOI:** 10.1016/j.ceb.2025.102521

**Published:** 2025-04-26

**Authors:** Maxx Swoger, Minh Tri Ho Thanh, Alison E. Patteson

**Affiliations:** 1 Department of Physics, Syracuse University, USA; 2 BioInspired Institute, Syracuse University, USA; 3 Department of Medicine, University of Pennsylvania, USA

## Abstract

Cells must navigate crowded and confining 3D environments during normal function *in vivo*. Essential to their ability to navigate these environments safely and efficiently is their ability to mediate and endure both self-generated and external forces. The cytoskeleton, composed of F-actin, microtubules, and intermediate filaments, provides the mechanical support necessary for force mediation. The role of F-actin and microtubules in this process has been well studied, whereas vimentin, a cytoplasmic intermediate filament associated with mesenchymal cells, is less studied. However, there is growing evidence that vimentin has functions in both force transmission and protection of the cell from mechanical stress that actin and microtubules cannot fulfill. This review focuses on recent reports highlighting vimentin’s role in regulating forces in confining environments.

## Introduction

The cytoskeleton, composed of F-actin, microtubules, and intermediate filaments, provides mechanical support to the cell and allows it to generate its own forces. Intermediate filaments are notable for their resilience to mechanical load in a variety of contexts, including gels composed of reconstituted proteins [1–3], single filaments [3,4], and inside of cells [5–7], suggesting that they likely have a role in providing mechanical resilience to cells navigating 3D environments. The role of epithelial keratin intermediate filaments in resisting mechanical stress is well known from studies of diseases associated with mutations in keratin or the proteins that link it to the cell membrane, but a function for the mesenchymal intermediate filament, vimentin, in cells in 3D environments is less well documented.

Vimentin has been implicated as an important factor for 2D mesenchymal migration in a number of ways. Vimentin regulates both focal adhesion dynamics [8,9] and actomyosin-generated traction stresses [10,11] while enabling cell persistence through templating microtubules as they undergo cycles of regrowth, conserving overall cell polarity [12]. During cell spreading and adhesion, vimentin deforms the cell nucleus, suggesting that vimentin transduces mechanical force onto the nuclear envelope [13,14]. The cytoskeleton is linked to the nucleus through the linker of nucleoskeleton and cytoskeleton (LINC) complex [15,16], and vimentin is cross-linked to actin through plectin [17] and to microtubules as cargo for motor proteins [18]. When cells migrate through 3D confining environments, the nucleus can be squeezed to the point of large deformations, blebbing, and rupture. Furthermore, it has been shown that the nucleus is mechanosensitive [19–22] and that forces can change DNA condensation states [23]. Recent work shows that vimentin protects the nucleus during 3D migration [24–27]. Vimentin serves as a mechanical link as cells migrate in both 2D and 3D environments, acting as a conduit for forces to be applied to the cell nucleus.

**Figure 1:**
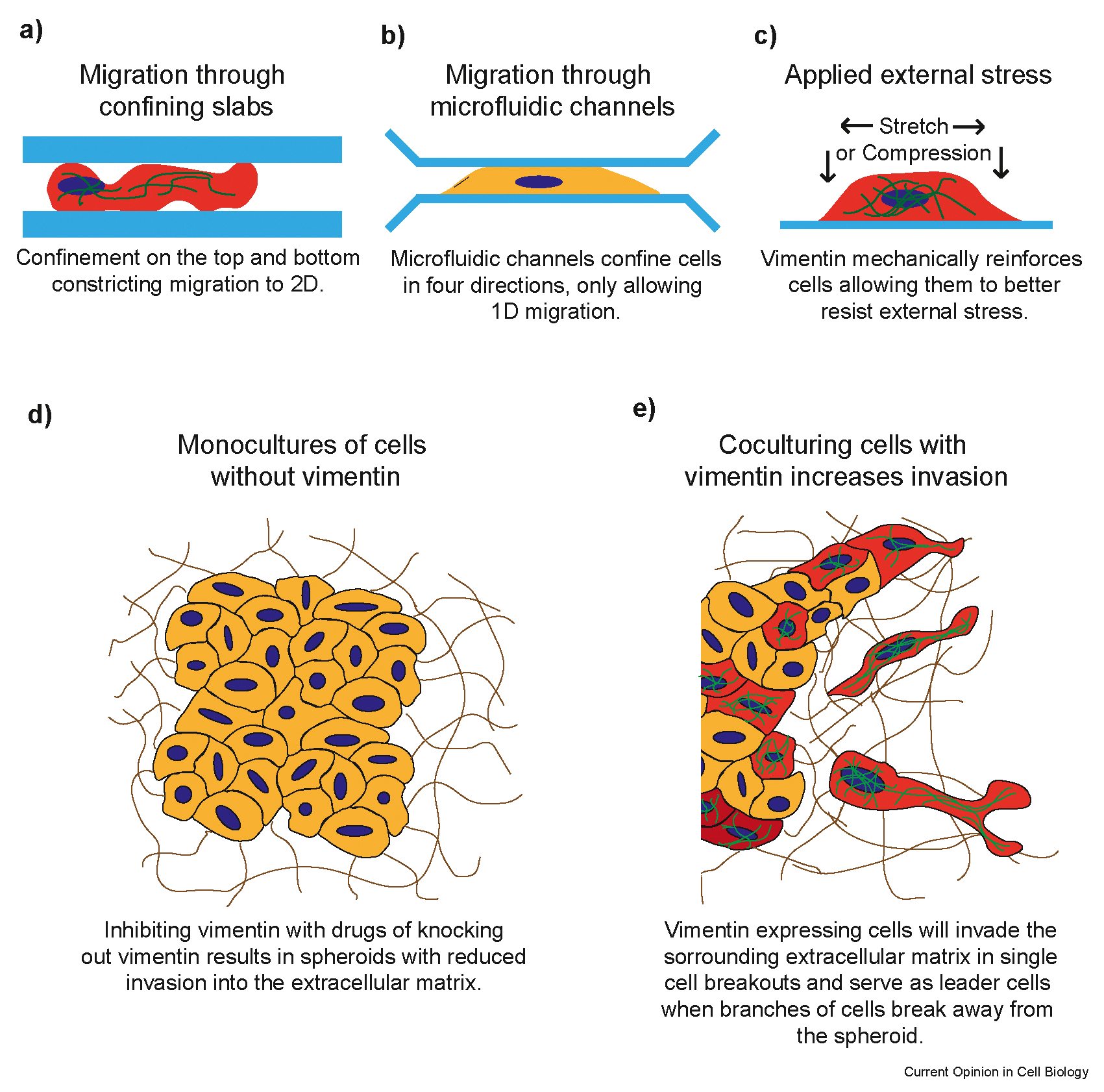
Schematics detailing systems used to study vimentin in 3D confining environments. Vimentin-expressing cells are colored orange, cells that do not express vimentin are colored yellow, cell nuclei are blue, confining geometries are light blue, vimentin is green, and the extracellular matrix is brown. a) shows migration between two confining slabs, b) shows migration through microfluidic channels, c) shows cells with an external stress or strain applied to them, d) shows a monoculture of vimentin deficient cells embedded in extracellular matrix exhibiting deficient invasion, and e) sketches a co-culture of cells with and without vimentin, where vimentin-expressing cells invade individually and as leader cells for cells without vimentin.

In this review, we discuss recent reports showcasing the role of vimentin in mediating forces in 3D confining environments. First, we focus on cells’ ability to migrate when confined between glass slabs (Figure 1a), microfluidic channels (Figure 1b), or on micropatterned substrates. Here we highlight how vimentin affects force transmission to the nucleus and new findings for the role of vimentin in amoeboid migration during confinement. Next, we discuss experiments where an external stress is introduced to cells and vimentin’s role in the response (Figure 1c), and how the response is a function of stress rate in addition to stress load. Lastly, we discuss how vimentin affects migration through physiologically relevant confining environments such as extracellular matrix hydrogels and tissue, particularly focusing on the effects of vimentin on invasion into the extracellular matrix both *in vitro* and *in vivo* (Figure 1d–e).

## The role of vimentin in 2D environments with external confinement

Studies of cells in 3D environments, such as within tissues or 3D extracellular matrices can be difficult. However, models can be designed based on 2D cell culture techniques with added external confinement to simulate a 3D environment. To this end, microfluidic channels, geometric confinement with micropatterns, and confinement between two glass slabs serve as simulated 3D environments. Furthermore, cells can be subjected to physiologically relevant external forces to probe the role of the cytoskeleton in mechanical response. Figure 1a–c show schematics of these experimental systems.

Microfluidic devices featuring confining microchannels or pores simulate the narrow spaces a cell would have to squeeze through when navigating the extracellular environment. In both mouse embryonic fibroblasts (mEFs) [24–26] and 3T3 fibroblasts [27], vimentin limits migration through confining spaces, as a result protecting the nucleus from undergoing excessive strain that can cause blebbing and rupture. Vimentin also limits the migration of glioblastoma cells as they attempt to migrate through microfluidic channels smaller than 5 microns in diameter [28]. Studies of cells migrating through small pores or microfluidic channels indicate that vimentin would likely inhibit migration through a 3D extracellular matrix. However, reports have shown that vimentin enhances invasion into the matrix [29]. Taken together, this suggests that vimentin regulates migration through confining spaces; however, in a more physiologically relevant situation, vimentin influences other factors for invasion, as discussed in a later section.

Geometric confinement using micropatterning, where a cell spread on a 2D substrate is restrained in area and shape, presents another technique that simulates a 3D environment. MEFs expressing vimentin spread on a high aspect ratio micropattern that simulates a cell undergoing highly polarized migration, such as stretching along a bundle of extracellular matrix, exhibited more nuclear deformation than cells lacking vimentin. Furthermore, knocking out nesprin-3, a mechanical link between vimentin and the nuclear envelope shows that this link is necessary for enhanced deformations [30]. Previous results have shown that external stress applied to the nucleus causes a contractile response in cells [31]. Vimentin might enhance the sensitivity of the cell nucleus to the extracellular environment by regulating force transmission to the nucleus, in turn affecting nuclear mechanosensitivity. This effect may help cells determine if a constricting pore is too small to safely migrate through, explaining why depleting vimentin results in enhanced migration through microchannels to the detriment of nuclear integrity. Furthermore, vimentin would contribute to cells’ ability to safely and efficiently navigate the extracellular matrix, suggesting why expression of vimentin may contribute to enhanced invasion, discussed later in this paper.

Amoeboid migration is a migration mode associated with movement through 3D confining environments where cells undergo rapid single-cell crawling, where adhesion forces play less of a role than in mesenchymal migration [32]. Recent studies have focused on the role of vimentin in amoeboid migration, with conflicting results. Depleting vimentin was shown to increase the migration of melanoma cells between glass slabs [33] but decrease migration of dendritic cells navigating in both microchannels and between glass slabs [34]. These conflicting trends may arise from differences in cell type or function; however, another study highlights the complex relationship between lamin-A/C and vimentin expression directly affecting amoeboid migration in confinement. Here, the authors use HeLa cells to show that lowering the expression of vimentin results in lower lamin A/C levels; conversely, lowering lamin A/C downregulates vimentin. The authors suggest that this feedback loop is to regulate the nuclear stiffness required for efficient amoeboid migration [35]. The role of vimentin in amoeboid migration may be an important function of overall cell stiffness and nuclear deformations regulated by vimentin, which are needed for the large cellular deformations required for amoeboid migration.

## Reactions to external applied stress

**Figure 2:**
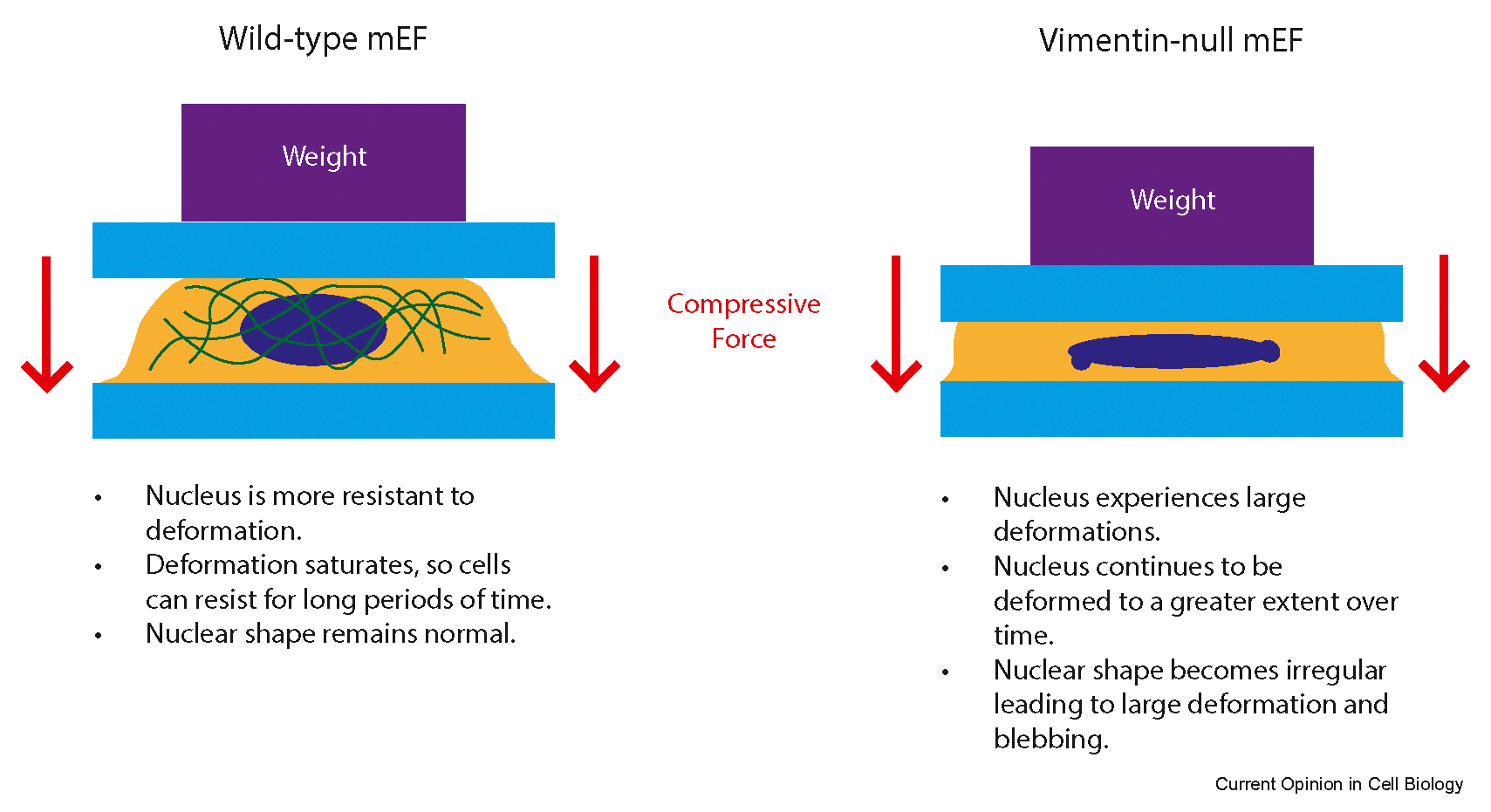
Vimentin reinforces the nucleus, resisting deformation at high compressive loads and over long periods of time. Confining coverslips are shown in blue, cells are covered yellow, nuclei are blue, vimentin is green, and weights applying pressure are purple. Cells without vimentin show more deformed nuclei when subjected to the same amounts of pressure and time of compression as cells expressing vimentin; furthermore, they exhibit irregular nuclear shape when under compressive load.

Cells *in vivo* are not only constrained by their environment, but external forces provide additional mechanical stresses to which the cell’s response depends on its stiffness as well as on the processes it subsequently activates. Previous studies have highlighted the role of vimentin in increasing the cells’ overall elastic stiffness, especially at large deformations [6,36,37]. A focus of recent studies has been on how the cell adapts to external stresses that cause nuclear deformation, which is of interest due to the mechanosensitive nature of the nucleus [23]. Vimentin resists compressive loading on the nucleus that can cause blebbing or rupture. Cells lacking vimentin show larger nuclear deformations when compressed with increasing compressive loads, and as a compressive load is held for a long period of time (Figure 2) [38]. These results suggest that vimentin may also provide mechanical support not just for high compressive loads, but also over long-term exposure to external stress.

Cells *in vivo* are exposed not just to a variety of strain levels but also to a variety of strain rates, and how vimentin responds to stress is a function of time scales. Glioblastoma cells expressing vimentin showed significant resistance to mechanical deformation when compressed with a single-cell microplate rheometer and intracellular rheology [28]. Vimentin links to fibrillar adhesions, long-lived adhesions colocalized with fibronectin fibrils, through plectin 1f, and dissipates stress from stretching cells to 110% of their original size [39]. In contrast, as cells spread on their substrates, they often make highly polarized cell shapes that add cell-generated strain to organelles like the nucleus over a long time. Vimentin aids cells in their response to mechanical perturbations by mediating force transmission to other parts of the cell, particularly the nucleus. Integral to this function are the physical links between vimentin and both the nuclear envelope and adhesions. A mechanical model for cell spreading that incorporates vimentin shows that vimentin will either reinforce or resist actomyosin and microtubule-based forces depending on substrate stiffness [40]. This model suggests a mechanism in which vimentin mechanically balances internal forces in a complex manner depending on environmental conditions, and this complexity is likely also present in how strain rate would affect how vimentin abates or enhances stress transfer. As a cell navigates in a 3D environment *in vivo*, it will experience a wide range of stresses at different rates; understanding how vimentin allows cells to adapt to these different conditions in a controlled 2D environment could provide insight into how cells function *in vivo*.

## The role of vimentin in navigating 3D environments

Numerous studies have implicated vimentin in 2D migration: vimentin supports adhesion-dependent migration [41], enhances cell polarity [12], modulates cell traction, and sustains cell–cell contacts during collective migration [42]. Unlike migration in 2D, cell migration in 3D often involves natural confining environments. Thus, experiments with cells in collagen gels, tissue assays, and *in vivo* models allow us to see how cells mediate forces in confining environments and translate to migration in a more physiologically relevant context.

Vimentin’s crucial role in driving cell invasion in 3D environments is demonstrated in recent studies. Förster resonance energy transfer (FRET) imaging of human foreskin fibroblasts revealed a strong interaction between actin and vimentin during single-cell migration, which decreased upon ROCK (rho kinase) inhibitor treatment in 3D cell-derived matrix [43]. Experiments involving vimentin in 3D collagen gels show that mEF spheroids [44] and lung cancer spheroids from a genetically engineered mouse model (GEMM) [45] significantly reduced their invasive capacity when vimentin is knocked out. Vimentin knockdown blocked the invasion of triple-negative breast cancer (TNBC) GEMM organoids and patient-derived xenograft organoids in a 3D collagen gel [46]. Similarly, in 3D Matrigel, knocking out vimentin in U251 glioblastoma (GBM) spheroids strongly decreased cell outgrowth [28]. Intriguingly, spheroids of MV3 melanoma cells (high vimentin, low E-cadherin expression) are more invasive than spheroids of A549 lung carcinoma cells (low vimentin, high E-cadherin expression) [47].

In animal model studies, injection of vimentin-depleted GBM cells into the zebrafish brain showed a markedly lower invasion index compared to control cells [28]. In orthotopic metastasis assays, vimentin knockdown significantly reduced both macrometastases (>100 cells) and micrometastases (<100 cells) in TNBC organoids [46]. However, in tail vein-based experimental metastasis, a significant increase in the number of macrometastases in vimentin knockdown groups is observed *in vivo*. This contradiction in the number of macrometastases in GEMM models, patient samples, and tail-vein metastasis model might be explained by selective pressures in distant organs that favor re-expression of vimentin.

During 3D invasion, cells often organize into chains, where leader cells drive the invasion, followed by follower cells. [48]. The observed invasiveness in vimentin-expressing cells indicates that vimentin promotes leader cell-directed collective migration, which has been shown previously in 2D wound healing experiments [29,42,49]. In GBM intermediate filament (IF) depleted cells, both leader and follower cells were significantly reduced, indicating that IFs play a critical role in maintaining the chain structure for invasion. In coculture spheroids, adding control (IF-expressing) cells increased the number of followers, showing that IF-expressing cells enhance invasion dynamics [28]. Organoids from TNBC patients were predominantly (>90%) composed of E-cadherin-positive/vimentin-positive (Ecad+/Vim+) cells leading invasive strands in 3D culture assays established from three different TNBC patient tumors [45].

Cell invasion in a 3D extracellular enviroment (ECM) relies on cell deformation through small spaces and active ECM degradation and remodeling [50], which is usually linked to matrix metalloproteinases (MMP) [51,52]. IF-depleted leader cells showed more nuclear deformations than control leader cells, suggesting that cytoplasmic IFs are predominant in protecting the nucleus from the compressive forces during invasion [28]. Nuclear deformation has been shown to cause localization of the protein YAP, a mechanosensitive signal molecule, to the nucleus [53]. Nuclear Yes-associated protein (YAP) translocation was increased in IF-depleted leader cells compared to control leader cells. MMP depletion, either through treatment of MMP inhibitor or shRNA, strongly reduced the invasion of control cells to a level similar to that of vimentin-depleted cells [28,44]. Furthermore, Mmp11, Mmp15, and Mmp24 have been observed to be significantly upregulated in vimentin +/+ cells [45]. Interestingly, Ho Thanh et al. showed that vimentin-depleted cells can invade laser-ablated micro-tracks next to the spheroid inside a collagen gel [44], highlighting the importance of vimentin and MMP interaction in collagen degradation. Moreover, collagen degradation is mechanically sensitive, as evidenced by experiments showing that mechanical compression via dextran reduces collagen degradation by invading vimentin wild-type cells [28,54]. An intact vimentin network is needed for the invasiveness observed, not just vimentin expression. mEF cells that only express vimentin unit-length-filaments (ULFs) exhibit a similar phenotype to vimentin KO mEFs in 3D collagen gels [44], and they failed to form lung tumors in an allograft tumor model in mice [45].

**Figure 3:**
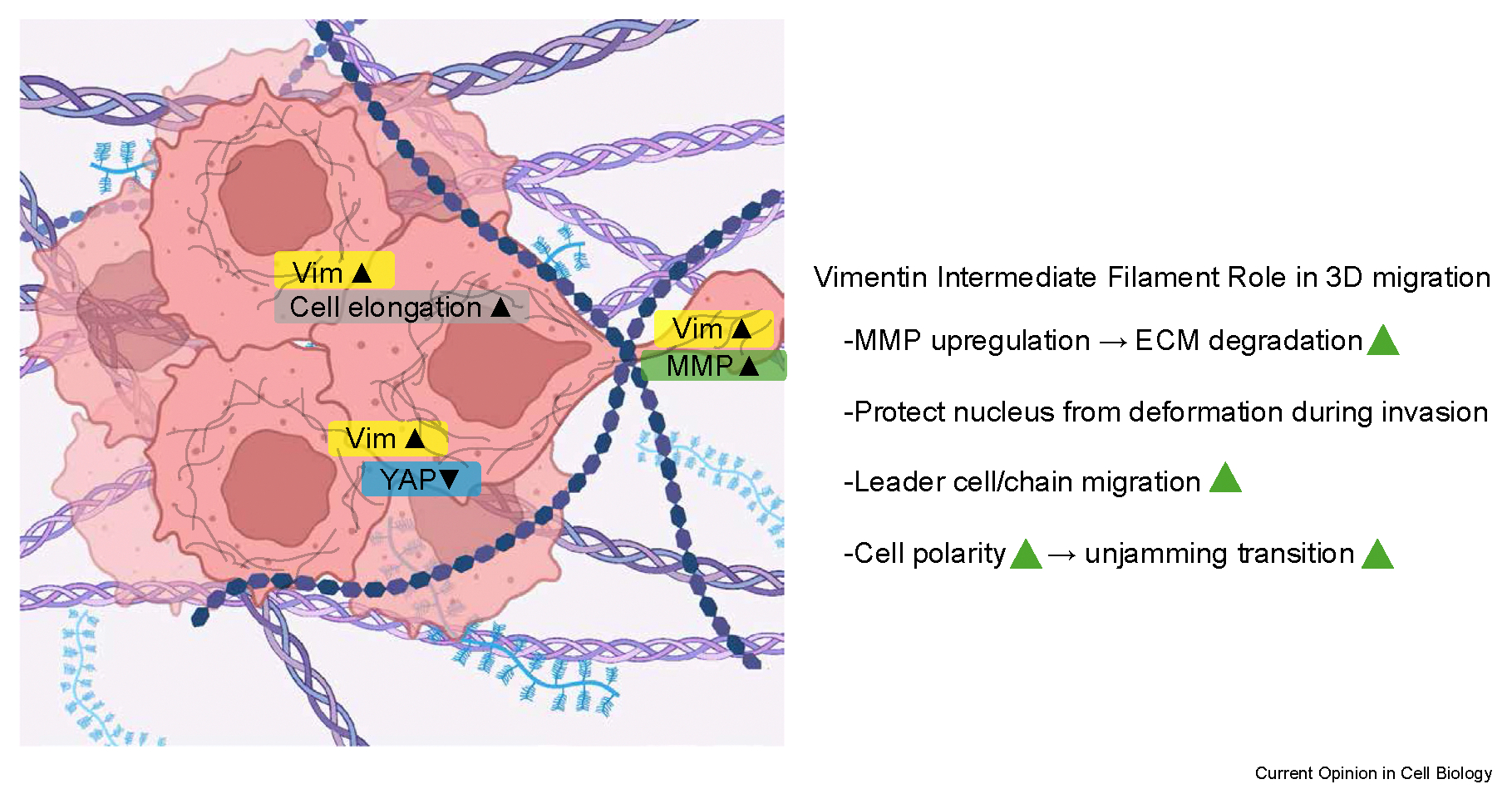
Mechanisms of vimentin intermediate filament enhancing collective cell migration in a 3D environment. 1) MMP is upregulated in cells with vimentin (Vim) intermediate filaments, thereby increases extacellular matrix degradation and remodeling. 2) Vimentin intermediate filaments protects the nucleus from compressive force during 3D migration, evidenced by nuclear Yes-associated protein (YAP) translocation increased in Vim −/− cells. 3) Vimentin promotes leader cell-directed collective migration. 4) Vimentin enhances cell elongation during migration, thus supports unjamming transition, promoting cells to leave aggregates. MMP, matrix metalloproteinases.

Lastly, spheroid invasion into the surrounding space also relies on an unjamming transition, where cells shift from a solid-like to a liquid-like state in densely packed environments [55–57]. This process involves cells elongating their shapes, moving past each other, and exerting traction forces onto the ECM to leave the cell aggregates, which also restructures the ECM network. van der Net et al. demonstrated the unjamming transition of cancerous spheroids depends on ECM density and MMP expression levels [47]. In a 2D wound healing assay, previous studies reported that vimentin enhances cell polarity by templating microtubules [12,58]. In 3D collagen gels, growth factor-induced elongation was higher in wild-type cells than in vimentin-null human keratocytes cells [59]. Similarly, mEFs’ cell shape and collagen contraction have been shown in a 3D spheroid experiment [44] and simulation [60] to be higher in a wild-type compared to vimentin-null cells. These results show that vimentin promotes a more fluid-like spheroid, encouraging invasion into the surrounding ECM, correlating with higher MMP levels and cell shape change (Figure 3). These findings suggest that vimentin promotes cell migration in 3D confining environments, which may help promote wound healing *in vivo*.

## Conclusion

Cells must navigate confining environments during migration *in vivo*, where the cell is often exposed to large stresses. The ability of these cells to resist large deformations is central to them performing their cellular functions. Intermediate filaments provide important mechanical support, allowing cells to function in these environments, but they have far more influence on cell function, especially in 3D culture, than simply adding mechanical resistance. Intermediate filament proteins, especially vimentin, are emerging as an important and necessary component for invasion into the extracellular matrix for cancer cell assays in both collagen gels and animal models. IF expression promotes the unjamming transition of cell aggregates and MMP expression, thereby increasing ECM degradation and remodeling. The work presented in this article highlights the growing need to include intermediate filaments in models for force mediation in confining environments. Elucidating the role of vimentin in invasion may provide new avenues for addressing issues in cancer and disease.

## Data Availability

No data was used for the research described in the article.

## References

[1] Janmey PA, Euteneuer U, Traub P, Schliwa M: Viscoelastic properties of vimentin compared with other filamentous biopolymer networks. JCB (J Cell Biol) 1991, 113:155–160. 2007620 10.1083/jcb.113.1.155 PMC2288924

[2] Charrier EE, Janmey PA: Chapter two - mechanical properties of intermediate filament proteins. In Omary MB, Liem RKH. Methods in enzymology, vol. 568. Academic Press; 2016:35–57. 26795466 10.1016/bs.mie.2015.09.009 PMC4892123

[3] Lorenz C, Forsting J, Style RW, Klumpp S, Köster S: Keratin filament mechanics and energy dissipation are determined by metal-like plasticity. Matter 2023, 6:2019–2033. 37332398 10.1016/j.matt.2023.04.014 PMC10273143

[4] Schepers AV, Lorenz C, Nietmann P, Janshoff A, Klumpp S, Köster S. Multiscale mechanics and temporal evolution of vimentin intermediate filament networks, vol. 118. Proceedings of the National Academy of Sciences; 2021, e2102026118. 10.1073/pnas.2102026118 PMC8271578 34187892

[5] Guo M, Ehrlicher Allen J, Mahammad S, Fabich H, Jensen Mikkel H, Moore Jeffrey R, Fredberg Jeffrey J, Goldman Robert D, Weitz David A: The role of vimentin intermediate filaments in cortical and cytoplasmic mechanics. Biophys J 2013, 105: 1562–1568. 24094397 10.1016/j.bpj.2013.08.037 PMC3791300

[6] Hu J, Li Y, Hao Y, Zheng T, Gupta SK, Parada GA, Wu H, Lin S, Wang S, Zhao X, : High stretchability, strength, and toughness of living cells enabled by hyperelastic vimentin intermediate filaments. Proc Natl Acad Sci 2019, 116: 17175–17180. 31409716 10.1073/pnas.1903890116 PMC6717279

[7] Nagle I, Delort F, Hénon S, Wilhelm C, Batonnet-Pichon S, Reffay M: The importance of intermediate filaments in the shape maintenance of myoblast model tissues. Elife 2022, 11, e76409. 36453730 10.7554/eLife.76409 PMC9754632

[8] Ostrowska-Podhorodecka Z, Ding I, Lee W, Tanic J, Abbasi S, Arora PD, Liu RS, Patteson AE, Janmey PA, McCulloch CA: Vimentin tunes cell migration on collagen by controlling β1 integrin activation and clustering. J Cell Sci 2021, 134. 10.1242/jcs.254359 33558312

[9] Gregor M, Osmanagic-Myers S, Burgstaller G, Wolfram M, Fischer I, Walko G, Resch GP, Jörgl A, Herrmann H, Wiche G: Mechanosensing through focal adhesion-anchored intermediate filaments. FASEB J 2014, 28:715–729. 24347609 10.1096/fj.13-231829

[10] Jiu Y, Lehtimäki J, Tojkander S, Cheng F, Jäälinoja H, Liu X, Varjosalo M, Eriksson John E, Lappalainen P: Bidirectional interplay between vimentin intermediate filaments and contractile actin stress fibers. Cell Rep 2015, 11:1511–1518. 26027931 10.1016/j.celrep.2015.05.008

[11] Costigliola N, Ding L, Burckhardt CJ, Han SJ, Gutierrez E, Mota A, Groisman A, Mitchison TJ, Danuser G. Vimentin fibers orient traction stress, vol. 114. Proceedings of the National Academy of Sciences; 2017:5195–5200. 10.1073/pnas.1614610114 PMC5441818 28465431

[12] Gan Z, Ding L, Burckhardt Christoph J, Lowery J, Zaritsky A, Sitterley K, Mota A, Costigliola N, Starker Colby G, Voytas Daniel F, : Vimentin intermediate filaments template microtubule networks to enhance persistence in cell polarity and directed migration. Cell Syst 2016, 3:252–263.e258. 27667364 10.1016/j.cels.2016.08.007 PMC5055390

[13] Sarria AJ, Lieber JG, Nordeen SK, Evans RM: The presence or absence of a vimentin-type intermediate filament network affects the shape of the nucleus in human SW-13 cells. J Cell Sci 1994, 107:1593–1607. 7962200 10.1242/jcs.107.6.1593

[14] Terriac E, Schütz S, Lautenschläger F: Vimentin intermediate filament rings deform the nucleus during the first steps of adhesion. Front Cell Dev Biol 2019, 7. 31263698 10.3389/fcell.2019.00106 PMC6590062

[15] Alam S, Lovett DB, Dickinson RB, Roux KJ, Lele TP: Chapter eight - nuclear forces and cell mechanosensing. In Engler AJ. Progress in molecular biology and translational science, vol. 126. Kumar S: Academic Press; 2014:205–215. 10.1016/B978-0-12-394624-9.00008-7 PMC4261915 25081619

[16] Vahabikashi A, Sivagurunathan S, Nicdao FAS, Han YL, Park CY, Kittisopikul M, Wong X, Tran JR, Gundersen GG, Reddy KL, . Nuclear lamin isoforms differentially contribute to LINC complex-dependent nucleocytoskeletal coupling and whole-cell mechanics, vol. 119. Proceedings of the National Academy of Sciences; 2022, e2121816119. 10.1073/pnas.2121816119 PMC9170021 35439057

[17] Favre B, Schneider Y, Lingasamy P, Bouameur J-E, Begré N, Gontier Y, Steiner-Champliaud M-F, Frias MA, Borradori L, Fontao L: Plectin interacts with the rod domain of type III intermediate filament proteins desmin and vimentin. Eur J Cell Biol 2011, 90:390–400. 21296452 10.1016/j.ejcb.2010.11.013

[18] Prahlad V, Yoon M, Moir RD, Vale RD, Goldman RD: Rapid movements of vimentin on microtubule tracks: kinesin-dependent assembly of intermediate filament networks. JCB (J Cell Biol) 1998, 143:159–170. 9763428 10.1083/jcb.143.1.159 PMC2132817

[19] Wang N, Tytell JD, Ingber DE: Mechanotransduction at a distance: mechanically coupling the extracellular matrix with the nucleus. Nat Rev Mol Cell Biol 2009, 10:75–82. 19197334 10.1038/nrm2594

[20] Navarro AP, Collins MA, Folker ES: The nucleus is a conserved mechanosensation and mechanoresponse organelle. Cytoskeleton 2016, 73:59–67. 26849407 10.1002/cm.21277

[21] Shen Z, Niethammer P: A cellular sense of space and pressure. Science 2020, 370:295–296. 33060351 10.1126/science.abe3881

[22] Murakami M, Nakatani Y, Kuwata H, Kudo I: Cellular components that functionally interact with signaling phospholipase A(2)s. Biochim Biophys Acta 2000, 1488:159–166. 11080685 10.1016/s1388-1981(00)00118-9

[23] Damodaran K, Venkatachalapathy S, Alisafaei F, Radhakrishnan AV, Sharma Jokhun D, Shenoy VB, Shivashankar GV: Compressive force induces reversible chromatin condensation and cell geometry–dependent transcriptional response. Mol Biol Cell 2018, 29:3039–3051. 30256731 10.1091/mbc.E18-04-0256 PMC6333178

[24] Patteson AE, Pogoda K, Byfield FJ, Mandal K, Ostrowska-Podhorodecka Z, Charrier EE, Galie PA, Deptuła P, Bucki R, McCulloch CA, : Loss of vimentin enhances cell motility through small confining spaces. Small 2019, 15, 1903180. 10.1002/smll.201903180 PMC6910987 31721440

[25] Patteson AE, Vahabikashi A, Pogoda K, Adam SA, Mandal K, Kittisopikul M, Sivagurunathan S, Goldman A, Goldman RD, Janmey PA: Vimentin protects cells against nuclear rupture and DNA damage during migration. JCB (J Cell Biol) 2019, 218:4079–4092. 31676718 10.1083/jcb.201902046 PMC6891099

[26] Gupta S, Patteson AE, Schwarz JM: The role of vimentin–nuclear interactions in persistent cell motility through confined spaces. New J Phys 2021, 23, 093042. 10.1088/1367-2630/ac2550 PMC9075336 35530563

[27] Zhou Z, Cui F, Wen Q, Susan Zhou H: Effect of vimentin on cell migration in collagen-coated microchannels: a mimetic physiological confined environment. Biomicrofluidics 2021, 15. https://pasteur.hal.science/pasteur-04257830/. 10.1063/5.0045197 PMC8133791 34025897

[28] van Bodegraven E J, Pereira D, Peglion F, Infante E, Kesenci Y, Terriac E, Geay J, Roca V, Plays M, Soto L, : Intermediate filaments promote glioblastoma cell invasion by controlling cell deformability and mechanosensitive gene expression. HAL Open Sci 2023. pasteur-04257830. 10.1016/j.celrep.2025.116553 41241939

[29] Bleaken BM, Menko AS, Walker JL: Cells activated for wound repair have the potential to direct collective invasion of an epithelium. Mol Biol Cell 2016, 27:451–465. 26658613 10.1091/mbc.E15-09-0615 PMC4751597

[30] Swoger M, Tri Ho Thanh M, Byfield FJ, Dam VB, Williamson J, Frank B, Hehnly H, Conway D, Patteson AE: Vimentin molecular linkages with nesprin-3 enhance nuclear deformation by cell geometric constraints. bioRxiv 2024, 2024. 2010.2029.621001. 10.1038/s41598-026-52647-9 PMC13396528 42168662

[31] Lomakin AJ, Cattin CJ, Cuvelier D, Alraies Z, Molina M, Nader GPF, Srivastava N, Sáez PJ, Garcia-Arcos JM, Zhitnyak IY, : The nucleus acts as a ruler tailoring cell responses to spatial constraints. Science 2020, 370. 33060332 10.1126/science.aba2894 PMC8059074

[32] Lämmermann T, Sixt M: Mechanical modes of ‘amoeboid’ cell migration. Curr Opin Cell Biol 2009, 21:636–644. 19523798 10.1016/j.ceb.2009.05.003

[33] Lavenus SB, Tudor SM, Ullo MF, Vosatka KW, Logue JS: A flexible network of vimentin intermediate filaments promotes migration of amoeboid cancer cells through confined environments. J Biol Chem 2020, 295:6700–6709. 32234762 10.1074/jbc.RA119.011537 PMC7212622

[34] Shaebani MR, Stankevicins L, Vesperini D, Urbanska M, Flormann DAD, Terriac E, Gad AKB, Cheng F, Eriksson JE, Lautenschläger F: Effects of vimentin on the migration, search efficiency, and mechanical resilience of dendritic cells. Biophys J 2022, 121:3950–3961. 36056556 10.1016/j.bpj.2022.08.033 PMC9675030

[35] Wang Y-J, Liang H, Liu Y, Bao Q, Yang S, Xu X-X, Chen Y-C, Liu W, Shi X, Shi Y, : Lamin A/C and vimentin as a coordinated regulator during amoeboid migration in microscale confined microenvironments. Nano Lett 2023, 23:6727–6735. 37459599 10.1021/acs.nanolett.3c02096

[36] Mendez MG, Restle D, Janmey PA: Vimentin enhances cell elastic behavior and protects against compressive stress. Biophys J 2014, 107:314–323. 25028873 10.1016/j.bpj.2014.04.050 PMC4104054

[37] Eckes B, Dogic D, Colucci-Guyon E, Wang N, Maniotis A, Ingber D, Merckling A, Langa F, Aumailley M, Delouvée A, : Impaired mechanical stability, migration and contractile capacity in vimentin deficient fibroblasts. J Cell Sci 1998, 111:1897–1907. 9625752 10.1242/jcs.111.13.1897

[38] Pogoda K, Byfield F, Deptuła P, Ciesluk M, Suprewicz Ł, Skłodowski K, Shivers JL, van Oosten A, Cruz K, Tarasovetc E, : Unique role of vimentin networks in compression stiffening of cells and protection of nuclei from compressive stress. Nano Lett 2022, 22:4725–4732. 35678828 10.1021/acs.nanolett.2c00736 PMC9228066

[39] Beedle AEM, Jaganathan A, Albajar-Sigalés A, Yavitt FM, Bera K, Andreu I, Granero-Moya I, Zalvidea D, Kechagia Z, Wiche G, : Fibrillar adhesion dynamics govern the timescales of nuclear mechano-response via the vimentin cytoskeleton. bioRxiv 2023. 10.1038/s41563-026-02590-x PMC13322966 42056228

[40] Alisafaei F, Mandal K, Saldanha R, Swoger M, Yang H, Shi X, Guo M, Hehnly H, Castañeda CA, Janmey PA, : Vimentin is a key regulator of cell mechanosensing through opposite actions on actomyosin and microtubule networks. Commun Biol 2024, 7:658. 38811770 10.1038/s42003-024-06366-4 PMC11137025

[41] Terriac E, Coceano G, Mavajian Z, Hageman TAG, Christ AF, Testa I, Lautenschläger F, Gad AKB: Vimentin levels and serine 71 phosphorylation in the control of cell-matrix adhesions, migration speed, and shape of transformed human fibroblasts. Cells 2017, 6:2. 28117759 10.3390/cells6010002 PMC5371867

[42] De Pascalis C, Pérez-González C, Seetharaman S, Boëda B, Vianay B, Burute M, Leduc C, Borghi N, Trepat X, Etienne-Manneville S: Intermediate filaments control collective migration by restricting traction forces and sustaining cell–cell contacts. JCB (J Cell Biol) 2018, 217:3031–3044. 29980627 10.1083/jcb.201801162 PMC6122997

[43] Marks PC, Hewitt BR, Baird MA, Wiche G, Petrie RJ: Plectin linkages are mechanosensitive and required for the nuclear piston mechanism of three-dimensional cell migration. Mol Biol Cell 2022, 33, ar104. 35857713 10.1091/mbc.E21-08-0414 PMC9635290

[44] Thanh MTH, Poudel A, Ameen S, Carroll B, Wu M, Soman P, Zhang T, Schwarz JM, Patteson AE: Vimentin promotes collective cell migration through collagen networks via increased matrix remodeling and spheroid fluidity. bioRxiv 2024, 2024. 2006.2017.599259. 10.1038/s42003-026-10506-3 PMC13639091 42315712

[45] Berr AL, Wiese K, dos Santos G, Koch CM, Anekalla KR, Kidd M, Davis JM, Cheng Y, Hu Y-S, Ridge KM: Vimentin is required for tumor progression and metastasis in a mouse model of non–small cell lung cancer. Oncogene 2023, 42:2074–2087. 37161053 10.1038/s41388-023-02703-9 PMC10275760

[46] Grasset EM, Dunworth M, Sharma G, Loth M, Tandurella J, Cimino-Mathews A, Gentz M, Bracht S, Haynes M, Fertig EJ, : Triple-negative breast cancer metastasis involves complex epithelial-mesenchymal transition dynamics and requires vimentin. Sci Transl Med 2022, 14, eabn7571. 35921474 10.1126/scitranslmed.abn7571 PMC9801390

[47] van der Net A, Rahman Z, Bordoloi AD, Muntz I, ten Dijke P, Boukany PE, Koenderink GH: EMT-dependent cell-matrix interactions are linked to unjamming transitions in cancer spheroid invasion. bioRxiv 2024, 2024:2008. 593120. 10.1016/j.isci.2024.111424 PMC11665421 39717087

[48] Vilchez Mercedes SA, Bocci F, Levine H, Onuchic JN, Jolly MK, Wong PK: Decoding leader cells in collective cancer invasion. Nat Rev Cancer 2021, 21:592–604. 34239104 10.1038/s41568-021-00376-8

[49] Walker JL, Bleaken BM, Romisher AR, Alnwibit AA, Menko AS: In wound repair vimentin mediates the transition of mesenchymal leader cells to a myofibroblast phenotype. Mol Biol Cell 2018, 29:1555–1570. 29718762 10.1091/mbc.E17-06-0364 PMC6080657

[50] Friedl P, Wolf K: Tumour-cell invasion and migration: diversity and escape mechanisms. Nat Rev Cancer 2003, 3:362–374. 12724734 10.1038/nrc1075

[51] Liotta LA UP Thorgeirsson, Garbisa S: Role of collagenases in tumor cell invasion. Cancer Metastasis Rev 1982, 1:277–288. 6309368 10.1007/BF00124213

[52] Ostrowska-Podhorodecka Z, Ali A, Norouzi M, Ding I, Abbasi S, Arora PD, Wong THF, Magalhaes M, McCulloch CA: Vimentin-mediated myosin 10 aggregation at tips of cell extensions drives MT1-MMP-dependent collagen degradation in colorectal cancer. FASEB J 2023, 37, e23097. 37440280 10.1096/fj.202300672R

[53] Dupont S, Morsut L, Aragona M, Enzo E, Giulitti S, Cordenonsi M, Zanconato F, Le Digabel J, Forcato M, Bicciato S, : Role of YAP/TAZ in mechanotransduction. Nature 2011, 474:179–183. 21654799 10.1038/nature10137

[54] Grolleman J, van Engeland NCA, Raza M, Azimi S, Conte V, Sahlgren CM, Bouten CVC: Environmental stiffness restores mechanical homeostasis in vimentin-depleted cells. Sci Rep 2023, 13, 18374. 37884575 10.1038/s41598-023-44835-8 PMC10603057

[55] Ilina O, Gritsenko PG, Syga S, Lippoldt J, La Porta CAM, Chepizhko O, Grosser S, Vullings M, Bakker G-J, Starruß J, : Cell–cell adhesion and 3D matrix confinement determine jamming transitions in breast cancer invasion. Nat Cell Biol 2020, 22:1103–1115. 32839548 10.1038/s41556-020-0552-6 PMC7502685

[56] Kim JH, Pegoraro AF, Das A, Koehler SA, Ujwary SA, Lan B, Mitchel JA, Atia L, He S, Wang K, : Unjamming and collective migration in MCF10A breast cancer cell lines. Biochem Biophys Res Commun 2020, 521:706–715. 31699371 10.1016/j.bbrc.2019.10.188 PMC6937379

[57] Bi D, Yang X, Marchetti MC, Manning ML: Motility-driven glass and jamming transitions in biological tissues. Phys Rev X 2016, 6, 021011. 28966874 10.1103/PhysRevX.6.021011 PMC5619672

[58] Saldanha R, Ho Thanh MT, Krishnan N, Hehnly H, Patteson A: Vimentin supports cell polarization by enhancing centrosome function and microtubule acetylation. J R Soc Interface 2024, 21, 20230641. 38835244 10.1098/rsif.2023.0641 PMC11285968

[59] Miron-Mendoza M, Poole K, DiCesare S, Nakahara E, Bhatt MP, Hulleman JD, Petroll WM: The role of vimentin in human corneal fibroblast spreading and myofibroblast transformation. Cells 2024, 13:1094. 38994947 10.3390/cells13131094 PMC11240817

[60] Zhang T, Ameen S, Ghosh S, Kim K, Thanh M, Patteson AE, Wu M, Schwarz JM: Enhanced extracellular matrix remodeling due to embedded spheroid fluidization. bioRxiv 2024, 2024:586590. 2025. 10.1088/1367-2630/ade81e PMC12242829 40656459

